# Challenges in the care and treatment of patients with extreme obesity

**DOI:** 10.20945/2359-4292-2023-0335

**Published:** 2024-07-18

**Authors:** Matheo A. M. Stumpf, Marcio C. Mancini

**Affiliations:** 1 Hospital das Clínicas Faculdade de Medicina Universidade de São Paulo São Paulo SP Brasil Unidade de Obesidade, Divisão de Endocrinologia e Metabolismo, Hospital das Clínicas, Faculdade de Medicina, Universidade de São Paulo, São Paulo, SP, Brasil

**Keywords:** Bariatric surgery, GLP-1 receptor agonists, mortality, extreme obesity, anti-obesity drugs

## Abstract

Obesity is a prevalent chronic disease. The management of extreme obesity – i.e., body mass index (BMI) ≥ 50 kg/m^2^ or obesity class IV and V – is still far from ideal. Individuals with extreme obesity have a high risk of surgical complications, mortality, comorbidities, and reduced weight loss following bariatric surgery. Although lifestyle changes and anti-obesity medications are recommended for all patients with extreme obesity as adjuvants to weight loss, these measures are less effective than bariatric surgery. As a first step, sleeve gastrectomy or an inpatient very-low-calorie diet should be incentivized to enhance weight loss before definitive surgery. Although malabsorptive procedures lead to greater weight loss, they are associated with an increased risk of early complications and malnutrition. Nonstandard techniques employed in clinical trial protocols, such as transit bipartition, may be performed as they maintain a weight loss potency comparable to that of the classic duodenal switch but with fewer nutritional problems. Anatomical causes should be investigated in patients with postoperative suboptimal clinical response or recurrent weight gain. In these cases, the initiation of anti-obesity drugs, endoscopic therapies, or a conversion procedure might be recommended. More studies are needed to address the specific population of patients with extreme obesity, as their outcomes are expected to be distinct from those of patients with lower BMI.

## INTRODUCTION

Obesity is a complex and multifactorial disease that is common enough to constitute a serious medical and public health problem. Medical and surgical specialists are often challenged by pathophysiological changes associated with this disease. Overwhelming evidence indicates that obesity carries excess risks. Indeed, mortality rises sharply when the body mass index (BMI) surpasses 30 kg/m^2^, particularly with a concomitant central distribution of adipose tissue (1). Obesity has serious effects on respiratory, cardiovascular, digestive, and genitourinary systems, imposing barriers to progress in some diagnostic and therapeutic procedures (2).

Estimates indicate that over 0.5% of the adult population in the United States has a BMI ≥ 50 kg/m^2^ (obesity class IV and V, formerly called “super obesity” and “super super obesity”, but referred to in this article as “extreme obesity”) (3). These individuals carry more obesity-related comorbidities, have higher surgical risk and increased mortality, and report lower quality of life than individuals with a BMI of 40-50 kg/m^2^ (*i.e.*, class III obesity) (4,5).

We discuss herein the main challenges in the care of extreme obesity and review the literature on its treatment, focusing on drugs and surgical procedures. The authors’ personal perspectives and experiences on these topics are also provided.

### Management of extreme obesity with anti-obesity medications and lifestyle changes

Individuals with obesity derive significant clinical benefits from a 5%-10% weight loss (6). However, this amount of weight loss is insufficient for people with extreme obesity. An increased weight loss achieved by these individuals results in a dose-dependent change in metabolic and mechanical comorbidities that leads to incremental clinical benefits. However, to date, there have been no randomized clinical trials specifically targeting the clinical treatment of individuals with extreme obesity alone.

Retatrutide – a triple agonist of the glucose-dependent insulinotropic polypeptide (GIP), glucagon-like peptide-1 (GLP-1), and glucagon receptors – at a dose of 12 mg for 48 weeks has been recently associated with a remarkable 24.2% weight loss in adults with obesity (7). Interestingly, patients with BMI ≥ 35 kg/m^2^ had an even greater weight loss (26.5%) (7). Tirzepatide, a dual GIP and GLP-1 receptor agonist, at a dose of 15 mg for 72 weeks, has also been associated with important weight loss (20.9%) in people with obesity (8). Semaglutide, another anti-obesity medication, is a GLP-1 receptor agonist administered subcutaneously once weekly at a 2.4 mg dose. This regimen achieves weight loss below 20%, comparable to the results obtained with a daily 50 mg oral dose (9,10). In all these trials, the medications were offered in conjunction with lifestyle changes (physical activity and hypocaloric diet). However, the high cost of these drugs limits their widespread use in daily clinical practice.

Traditional anti-obesity medications (*e.g.*, sibutramine, naltrexone plus bupropion, topiramate, orlistat) lack the potency of the more recent agonist drugs. However, due to their relatively low cost and extensive literature experience, off-label combinations of these traditional medications could lead to substantial weight loss (11), providing an option for adjuvant therapy in individuals with extreme obesity. Additionally, treatment with these traditional anti-obesity drugs may be attempted in patients with eating disorders, as topiramate, lisdexamfetamine (the only FDA-approved medication for binge-eating disorder), and, more recently, GLP-1 agonists have been shown to improve binge-eating episodes (12). In syndromic obesity (*e.g.*, Prader-Willi syndrome), lisdexamfetamine also appears to have an effect on reducing hyperphagia episodes (13). However, reducing binge severity has proven to have little impact on weight loss (14), and its effect on extreme obesity should be modest.

In fewer than 5% of the patients with extreme obesity, an identifiable monogenic cause may be present (15), potentially guiding a different treatment approach. Monogenic etiologies should be considered in individuals with clinical features such as early-onset obesity (often involving children younger than 10 years), rapid onset of weight gain occurring before the age of 2 years, endocrine disorders (adrenal insufficiency, hypogonadism, short stature), and immune dysfunction (chronic infections, diarrhea) (16). The identification of monogenic causes is important since, depending on the mutation, treatment with a specific drug could almost normalize the individual’s BMI.

### Bariatric surgery as a therapeutic approach

#### Preoperative and perioperative care: bridging procedures and anesthesia

Due to its overall safety profile and strong association with weight loss, bariatric surgery should be considered the first-line treatment for people with extreme obesity. A lifetime procedure, bariatric surgery does not require a high level of patient compliance, ensuring long-term benefits (1). The use of bridging interventions before bariatric surgery is an interesting approach for patients with extreme obesity, as they do not experience continuous weight loss beyond the initial 12-month rapid weight loss phase, unlike patients with BMI < 50 kg/m^2^ (17).

A survey focusing on patients with extreme obesity, responded by 789 bariatric surgeons from 73 countries, found that 55.5% of the respondents encouraged weight loss before surgery, but just a few (3.6%) suggested the insertion of an intragastric balloon (IGB) (18). Sleeve gastrectomy (SG) was considered the best choice for patients younger than 18 years or older than 65 years. The most chosen surgical procedures for patients aged 18-65 years were SG and one-anastomosis gastric bypass (OAGB), although half of the surgeons responded that a two-stage approach, with SG as the first stage, should be offered to patients with extreme obesity (18). Unlike SG, which has consistent outcomes and is an attractive first-stage surgical procedure for extreme obesity (19), laparoscopic adjustable gastric band (LAGB) should not be used as a bridging intervention due to its overall poor results (20).

Bridging intervention with IGB is a matter of debate and controversy. In a recent meta-analysis, IGB was not associated with significant weight loss before bariatric surgery in patients with extreme obesity, unlike first-step laparoscopic SG and a liquid low-calorie diet program, which were associated with mean BMI reductions of 15.2 kg/m^2^ and 9.8 kg/m^2^, respectively (21). In another study, IGB resulted in a mean weight loss of 17.3 ± 14.1 kg (BMI reduction of 5.8 ± 4.7 kg/m^2^), with a nadir 5 months after the procedure (22). However, patients who were pretreated using this strategy experienced an attenuated postoperative weight loss, with an earlier nadir and earlier recurrent body weight gain (22). Importantly, weight regain can occur in the time interval (3-4 weeks) between the IGB removal and the bariatric surgery, which is an important period for the resolution of gastric inflammation, reduction of wall thickness, and wound healing (22,23).

**Figure 1:**
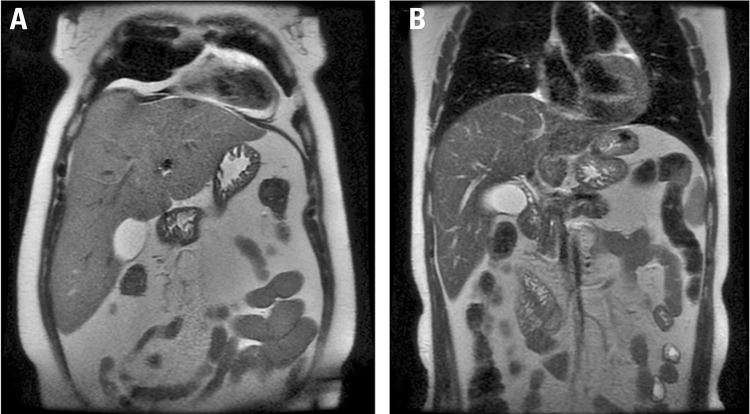
Abdominal magnetic resonance images (coronal plane) of a patient who underwent an inpatient very-low-calorie diet for 20 weeks. The images illustrate a remarkable reduction in liver size and in subcutaneous and visceral abdominal adipose tissue. (A) Before weight loss: weight 249 kg, body mass index (BMI) 77 kg/m2. (B) After a weight loss of 76 kg: weight 173 kg, BMI 54 kg/m2 (courtesy Prof. Dr. Marco Aurélio Santo).

Another strategy for patients who are unable to undergo IGB or SG as a first-step procedure is hospitalization for weight loss. With treatment on an inpatient basis, it is possible to maintain a controlled environment with a very-low-calorie diet, enhancing substantially the probability of achieving successful postoperative weight loss. In a retrospective analysis of 20 patients with extreme obesity hospitalized for a mean of 19.9 weeks, the achieved weight loss with a 5 kcal/kg/day diet was 19%, even in the absence of drug treatment or physical activity. No major surgical or postoperative complications were described in this high-risk group of patients (24). Specifically in the population with extreme obesity, greater reduction in body fat and fat-free mass has also been observed during a very-low-calorie diet (25). Figure 1 shows before and after abdominal magnetic resonance images of a patient who underwent an inpatient very-low-calorie diet for 20 weeks (losing 76 kg of weight – equivalent to 23 kg/m^2^ BMI, from 249 kg to 173 kg), a routine treatment performed for extreme obesity in our service.

The goal of preoperative hospitalization is not to achieve the greatest possible weight loss, but rather to attain sufficient weight reduction to minimize surgical risks. Ideally, bariatric surgery should be performed shortly after this weight loss since only one-third of the patients in an outpatient setting remain compliant and maintain weight losses ≥ 15 kg after 12 months (26).

During the preoperative period, a radiologic evaluation of the upper airway, as well as an otorhinolaryngologic consultation with direct or indirect laryngoscopy, can provide useful information about the patient’s airway (2). This is important as some intraoperative interventions, such as reverse Trendelenburg and prone position, may be beneficial in patients with narrow airways, leading to better oxygenation and lower risk of atelectasis and hypoxemia (27).

In general, patients with obesity – and particularly those with extreme obesity – require mechanical ventilation with a high fraction of inspired oxygen and, eventually, the addition of high positive end-expiratory pressure (2). A high respiratory rate and low tidal volume are also warranted due to reduced total respiratory compliance (28,29).

Other recommendations following bariatric procedures include opioid-sparing anesthesia (to avoid apnea) and the use of noninvasive ventilation following extubation. A useful approach for pain management is the use of local anesthetics and thoracic epidural analgesia in cases of laparotomy (30).

#### Head-to-head comparison of techniques and weight loss

In the last decades, important progress has been made worldwide in terms of bariatric surgery for the treatment of extreme obesity. Bariatric surgery is regarded as a procedure capable of eliminating (or, at least greatly improving) a disease that is resistant to conventional treatments, offering a more effective choice for long-term weight loss and also improving associated health conditions (31,32). Importantly, current guidelines – for example, those from the European Society of Endocrinology – recommend excluding the occurrence of hypercortisolism in patients with obesity before bariatric surgery, although this is not an evidence-based strategy (33).

An important issue concerns the most suitable surgical procedure for patients with extreme obesity. Some experts recommend biliopancreatic diversion (BPD) with duodenal switch (DS), Roux-en-Y gastric bypass (RYGB), or OAGB for these patients, while others advise a two-stage procedure, with SG as the primary stage, followed by BPD/DS, RYGB, or OAGB (34).

A retrospective review of 498 patients with extreme obesity who underwent SG, RYGB, or OAGB showed that SG and OAGB were safe and effective primary surgical procedures, and that weight loss was superior with OAGB and RYGB than SG (35). On the other hand, a study comparing RYGB, LAGB, and SG (36) found percentages of total weight loss (TWL) during the first year of 36.3%, 31.6%, and 21.1% respectively, favoring nonrestrictive techniques as best candidates for treating extreme obesity concerning exclusively weight loss (36,37). In another retrospective study including more than 500 patients with extreme obesity, the procedures with the greatest percentages of TWL were BPD/DS (38.4%), followed by RYGB (26.3%) and SG (23.6%) (38). Notably, the 30-day complication rate was significantly higher in the BPD/DS group (12.9%) compared with the RYGB (4.7%) and SG (8.7%) groups (38).

Recent systematic reviews and meta-analyses comparing RYGB and SG in patients with extreme obesity reported superior weight loss and a higher resolution of dyslipidemia and type 2 diabetes with RYGB (39,40).

Suggested alternatives for enhancing weight loss in patients with extreme obesity could involve the modification of standard procedures (41). Examples of such modifications include using a longer (150 cm) alimentary limb in RYGB (42); establishing a very short (100-150 cm) common channel where digestion and absorption occur along with a “very very” long alimentary limb (400-500 cm) in RYGB (43) or a longer biliopancreatic limb (44,45); and single anastomosis duodenoileal bypass with SG (SADI, also known as one-anastomosis DS) (46) or SG with transit bipartition (TB), both derived from BPD/DS, which maintain powerful weight loss with a lower risk of protein malnutrition (47,48).

A retrospective study (48) comparing TB and BPD/DS in patients with extreme obesity demonstrated that TB was faster to perform and was associated with shorter hospital stay, less malnutrition, and fewer diarrhea episodes. After 1 year of follow-up, there were similar rates of comorbidity improvement and slightly more weight loss in the BPD/DS group (TWL 45 ± 6.7% with BPD/DS *versus* 41.3 ± 7.5% with TB; BMI 30.1 ± 4.1 kg/m^2^ with BPD/DS *versus* 31.5 ± 4.8 kg/m^2^ with TB, p < 0.05) (48).

In Brazil, only LAGB, SG, RYGB, and BPD (Scopinaro’s surgery or DS) are authorized by the Federal Council of Medicine (49). Other techniques could be used in the setting of clinical studies upon approval by ethics committees.

Table 1 summarizes the main outcomes of head-to-head studies analyzing different treatment techniques for extreme obesity. As shown, some authors currently prefer treating extreme obesity with disabsorptive procedures to enhance loss of weight and control of comorbidities, although more complications can occur with these procedures (50).

**Table 1:** Summary of the main outcomes of head-to-head studies comparing different techniques for the treatment of extreme obesity

Weight loss	Resolution of comorbidities	Early outcomes* (within 30 days)	Late complications**
RYGB > LAGB (51,52)	-	RYGB = LAGB (51); RYGB > LAGB (52)	-
RYGB = OAGB > SG (35,53)	RYGB = OAGB > SG (35)	RYGB > OAGB = SG (35); SG > OAGB (53)	-
BPD/DS > RYGB (54-58) = SG (38,59)	BPD/DS > RYGB (55,60) = SG (59)	BPD/DS > RYGB = SG (38); BPD/DS = RYGB (54)	BPD/DS > RYGB (57) = SG (38); BPD/DS = RYGB (54)
BPD/DS = SADI (46)	BPD/DS = SADI (46)	BPD/DS = SADI (46)	-
BPD (Scopinaro) > LAGB (61)	-	BPD (Scopinaro) > LAGB (61)	-
BPD/DS > TB (48) > RYGB (62)	BPD/DS = TB (48) = RYGB (62)	TB = RYGB (62)	BPD/DS > TB (48)

*Includes longer hospital stay, length of surgery, bleeding or blood loss, and wound infection. **Includes malnutrition, daily stool frequency, anemia, and requirement for revisional surgery. “>” indicates that one procedure is superior to another in terms of treatment outcomes, while “=” indicates that the outcomes of the procedures are comparable. Abbreviations: BPD/DS, biliopancreatic diversion with duodenal switch; LAGB, laparoscopic adjustable gastric band; OAGB, one-anastomosis gastric bypass; RYGB, Roux-en-Y gastric bypass; SADI, single anastomosis duodenoileal bypass with sleeve gastrectomy; SG, sleeve gastrectomy; TB, sleeve gastrectomy with transit bipartition.

## Failure after bariatric surgery, recurrent weight gain, and revisional operations

The criterion defining failure after bariatric surgery as a loss of less than 50% excess weight loss (EWL) after the procedure was proposed more than 40 years ago (63). This definition remains widely used today, although bariatric procedures vary in terms of their effect on weight loss and comorbidity resolution. Many patients – especially those with extreme obesity – are unable to maintain an EWL of 50% or more in the long term and are thus considered to have a suboptimal clinical response (64). A study of patients with extreme obesity who underwent RYGB showed that more than 75% of them achieved an EWL > 50% 2 years after surgery (65). Another study demonstrated that, while individuals with a BMI ≥ 60 kg/m^2^ experienced less weight loss compared with those with a BMI < 60 kg/m^2^, the health and quality of life of all participants improved, regardless of their preoperative BMI (66). In such cases, a meaningful enhancement in quality of life and improvements in hypertension, diabetes mellitus, dyslipidemia, and other comorbidities could also be considered a sign of optimal clinical response (67). Either way, patients with a weight loss of less than 50% EWL should be further investigated for procedural failures, such as slippage of the gastric band, gastro-gastric fistulas, dilation of the gastric fundus, and enlargement of the gastric pouch or gastro-jejunal stoma. The most common causes of suboptimal clinical response or recurrent weight gain following bariatric surgery are thought to be alterations in eating behavior (*i.e.*, binge, grazing), noncompliance with lifestyle recommendations, and return to previous dietary habits. Psychiatric disorders, especially anxiety and depression, have also been implicated as potential causes of treatment failure (68).

No consensus has been established on the definition of recurrent weight gain after bariatric surgery. A recent position statement by the Brazilian Society of Bariatric and Metabolic Surgery classified recurrent weight gain as recidivism (when 50% of the weight lost is regained in the long term or 20% of the weight is regained in association with reappearance of comorbidities) or controlled recidivism (when 20%-50% of the weight lost is regained in the long term) (69). A long-term recurrent weight gain of less than 20% of the weight lost is expectable (69). A practical definition of recurrent weight gain is a weight increase of ≥ 10 kg (or > 10%-15%) from the nadir weight (70). The approach to patients with weight regain is similar to that of patients with suboptimal clinical response, who do not achieve a > 50% EWL or who have a maximum TWL outcome < 20%. Therefore, it is very important to regularly reevaluate the patient’s diet, cognitive-behavioral therapy, and physical activity, along with conducting anatomical assessments through upper gastrointestinal endoscopy and/or a contrast x-ray study (68).

In our service, the drug of choice for patients experiencing recurrent weight gain after bariatric surgery is topiramate – either alone or combined with sibutramine and/or orlistat (71,72). However, the weight loss achieved with this approach is modest, typically around 3-6 kg. Recently, the use of GLP-1 receptor agonists (liraglutide and semaglutide) following recurrent weight gain has shown results in weight loss very similar to those observed when these drugs are used as primary obesity treatments (73-75). This new evidence suggests that patients with weight regain should receive the same therapy as treatment-naïve ones, with potent modern drugs or combinations of traditional ones.

Interestingly, one study found greater weight loss when anti-obesity medication was initiated during the weight plateau phase compared with after the recurrent weight gain (76). Therefore, proactive medical therapy at the time of weight plateau can help patients achieve greater TWL.

Another strategy is the use of weight loss medication “prophylactically”, although this approach has not been tested in clinical trials (71,77). This may be appropriate in patients with extreme obesity and stable weight who remain with a high BMI even after substantial postoperative weight loss. A retrospective study of 63 patients who had undergone RYGB and were followed up for more than 10 years compared the outcomes between those with BMI < 50 *versus* ≥ 50 kg/m^2^. Notably, the BMI ≥ 50 kg/m^2^ (extreme obesity) group included 66.7% of all study patients. At 10 years, the mean BMI decreased from 44.2 kg/m^2^ to 34.8 kg/m^2^ in patients with baseline BMI < 50 kg/m^2^ (an EWL of 43.8%) and from 60.4 kg/m^2^ to 39.7 kg/m^2^ (an EWL of 53.9%) in those with baseline BMI ≥ 50 kg/m^2^ (78). Thus, patients with extreme obesity maintained a mean BMI close to class III obesity even after bariatric surgery. This finding supports the recommendation for early use of anti-obesity medication in patients with extreme obesity, as obesity is not cured and remains present, given its chronic nature.

In patients who do not respond to anti-obesity medications and have important recurrent weight gain, or when an anatomic cause for recurrent weight gain is identified, revisional bariatric surgery may be indicated. Excluding those procedures performed after LAGB, conversion procedures are generally associated with higher risks than those of the primary bariatric surgery. This occurs because the second surgery is executed on organs that have been previously operated on and are, therefore, marked by surgical staples, reduced vascularization, and greater susceptibility to adhesions and fibrosis. The same rationale partly explains the limited effectiveness of revision surgery, as the ideal technical settings (*i.e.*, pouch size, sleeve size) may not be achievable (79,80).

Most revision surgeries after LAGB consist of conversions to RYGB, SG, or OAGB. After SG, the most frequent conversions are to RYGB, OAGB, re-sleeve gastrectomy, or SADI (81). When RYGB is the primary surgery, several corrections can be proposed, including surgical pouch size reduction, prolongation of the biliopancreatic limb, and surgical stoma size reduction, although some surgeons choose an endoscopic approach (pouch or stoma size reduction) or conversion to BPD/DS or TB (82).

It should be noted that the weight loss achieved with endoscopic revisional procedures is similar to that obtained with traditional anti-obesity drugs. Endoscopic transoral outlet reduction of gastrojejunal anastomosis after RYGB has a TWL of approximately 10%. The same mean percentage has been observed following endoscopic revisional sleeve gastroplasty after SG (83,84). On the contrary, the conversion of restrictive techniques (LAGB or SG) into RYGB or BPD/DS has generally shown comparable weight outcomes to primary RYGB or BPD/DS, at the cost of more complications (80).

## Risks following surgical procedures

Despite the large number of comorbidities presented by candidates for bariatric surgery, the procedure can still be considered overall safe, with a mortality risk of approximately 0.8% (32). At our hospital, patients with extreme obesity represent approximately 40% of all patients undergoing bariatric surgery. This group of patients has an increased risk of complications and higher rates of suboptimal clinical response associated with their increased BMI. Additionally, extreme obesity is associated with a higher incidence of comorbidities, major technical challenges, increased risks of surgical and anesthetic complications, and more perioperative and postoperative adversities. In contrast, weight loss before surgery decreases the morbidity of these patients to levels comparable to those of patients with less severe obesity. A retrospective analysis of data from patients operated on 5 years before our preoperative weight loss program was implemented showed that patients with extreme obesity had an approximately fourfold higher incidence of complications compared with those with BMI < 50 kg/m^2^, accounting for 80% of the deaths (85).

The first risk scale specific for bariatric surgery – the Obesity Surgery Mortality Risk Score (OS-MRS) – was developed in 2007 based on a multivariate analysis of preoperative factors associated with mortality in more than 2,000 RYGB procedures (86). The OS-MRS defines five independent factors of mortality risk (*i.e.*, age ≥ 45 years, male sex, BMI ≥ 50 kg/m^2^, hypertension, and risk factors for pulmonary embolism), assigning one point for each factor. Patients are categorized according to scores into one of three groups: low risk (class A), 0-1 point; intermediate risk (class B), 2-3 points; and high risk (class C), 4-5 points (Table 2). Therefore, a male patient with a BMI ≥ 50 kg/m^2^ has a moderate risk; if hypertension and age ≥ 45 years are added, the patient is then categorized at high risk for mortality (*i.e.*, 10-times higher than the risk attributed to the low-risk group).

**Table 2:** Obesity Surgery Mortality Risk Score (OS-MRS) stratification according to clinical parameters*

Risk factors		Points
Hypertension		1
Age ≥ 45 years		1
Male sex		1
Body mass index ≥ 50 kg/m^2^		1
Risk factor for pulmonary embolism**		1
Risk group classification	Score	Postoperative mortality
A (low)	0-1	0.3%
B (moderate)	2-3	1.7%
C (high)	4-5	3.2%

*Adapted from reference 86. **At least one of the following: previous history of pulmonary thromboembolism or deep vein thrombosis, hypoventilation (partial pressure of carbon dioxide [PaCO2] ≥ 45 mmHg), diagnosis of pulmonary hypertension, or presence of inferior vena cava filter.

Bariatric surgery has the potential to trigger several complications, one of which is rhabdomyolysis. This complication is characterized by muscle lysis and necrosis because of sarcolemmal damage, causing the release of myoglobin and creatine phosphokinase (CPK) into the circulation. Patients with rhabdomyolysis may have serum CPK levels exceeding 1,000 U/L or greater than five times the normal value (87). If the diagnosis of rhabdomyolysis is delayed and appropriate treatment is not administered in time, serious complications can occur, including acute renal failure or even death. A recent meta-analysis identified a prevalence of rhabdomyolysis of almost 20% in patients undergoing bariatric surgery, which increased with the duration of the surgery. For individuals undergoing bariatric surgery lasting more than 180 minutes, those undergoing RYGB, and patients with extreme obesity, CPK levels could be routinely measured early after surgery to verify the presence of rhabdomyolysis and actively prevent its complications (88).

Venous thrombosis remains the main cause of readmission and mortality following bariatric surgery (89). For thromboprophylaxis, the choice of agent, along with its dose and duration of use, is currently controversial. In individuals with extreme obesity, measurement of anti-factor Xa level should be considered for dose optimization if enoxaparin or rivaroxaban is used (90), and the use of these medications should be considered for an extended period (2-4 weeks) (30).

Other guideline-based strategies should also be used to mitigate additional risks, although these strategies are not specifically addressed for patients with extreme obesity (30). These strategies include the use of a proton pump inhibitor for at least 30 days (to prevent marginal ulcers and gastroesophageal reflux) and ursodeoxycholic acid for 6 months (to prevent gallstones) (91,92), particularly after RYGB. Some authors opt for performing prophylactic cholecystectomy concurrently with malabsorptive procedures to prevent the formation of gallstones and cholelithiasis (48). However, most do not routinely recommend prophylactic cholecystectomy, especially when the gastrointestinal anatomy remains unchanged, for example, in patients undergoing SG and TB, in whom full endoscopic access to the biliary tree is maintained (93).

**Figure 2:**
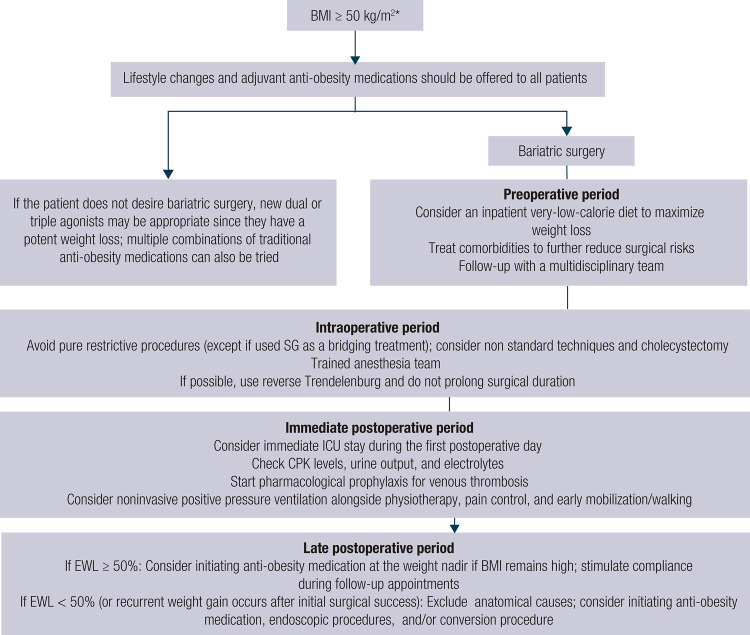
Suggested management approach for patients with extreme obesity.

In conclusion, extreme obesity is a challenging disease that can present with multiple comorbidities and high rates of mortality and complications following bariatric surgery. The flowchart in Figure 2 summarizes some of the main recommendations for the care of individuals with extreme obesity, even though its management is still far from state of the art. More studies should be conducted specifically in patients with this degree of obesity, since their outcomes are expected to be distinct from those of people with lower BMI.

*Consider genetic testing if extreme obesity occurs during childhood accompanied by clinical features such as adrenal insufficiency, hyperphagia, red hair, recurrent infections, or chronic diarrhea. Abbreviations: BMI, body mass index; CPK, creatine phosphokinase; EWL, excess weight loss; ICU, intensive care unit; SG, sleeve gastrectomy.
